# Supplementary material for: Feasibility and acceptability of daily testing at school as an alternative to self-isolation following close contact with a confirmed case of COVID-19: a qualitative analysis
Source: BMC Public Health. 2022 Apr 13;22:742. doi: 10.1186/s12889-022-13204-x (PMC9007577; doi:10.1186/s12889-022-13204-x)
Supplement: Supplementary file 1 — Additional file 1. [file 12889_2022_13204_MOESM1_ESM.docx]

**Experiences of daily coronavirus testing in schools – topic guide**

**Daily testing sample (students)**

I would like to start by asking you about your experiences of testing yourself for COVID-19.

- What happened when you were told that you had been in contact with someone with the virus?
- What made you decide to carry out daily testing at school instead of self-isolating?
- How did you feel when you found out that you could take tests for seven days instead of isolating?
- Did you have any concerns or worries about daily testing?
- Did you have any concerns or worries about self-isolating?

Experiences of testing

- Tell me about the first day, when you took the first of the seven tests. What happened?
- How does the seven day testing process differ to the twice weekly tests?
- What was it like taking the test?
- Were you happy with the information received/were you confident that you had taken the test correctly?
- What was the most difficult part of testing?
- Where there any days that you did not take a test?
- What happened on those days?

Behaviour during testing

- Did you/your teacher/class do anything differently at school during the seven days of testing?
- Did you/your household do anything differently at home during the seven days of testing?
- How did you feel about these changes?

Understanding and impact of test results

- How do you find out the results of your test?
- What (if any) information did receive with your test result? Was anything unclear / missing?
- What is your understanding of what your test result means?
- How did you feel when you received your test result?
- How did your result affect you? What were you able to do/not do?
- Did you have any concerns about what you could / could not do?
- Were there any times that you didn’t / or were unable to follow the rules?
- How did it affect your family/household? What were they able to do/not do?
- How confident were you that the results of the test were accurate?

Improvements

- Would you be willing to take daily tests instead of self-isolating again?
- What could be done to make it easier for people to take tests instead of isolating?
- What could be done to improve daily testing?

**Daily testing sample (parents)**

I would like to start by asking you about your experiences of daily testing for COVID-19.

- What happened when you were told that your child had been in contact with someone with the virus?
- What made you and your child decide to carry out daily testing at school instead of self-isolating?
- How did you feel when you found out that your child could take tests for seven days instead of isolating?
- Did you have any concerns or worries about daily testing?
- Did you have any concerns or worries about self-isolating?

Behaviour during testing

- Were you aware of any rules/ changes that had been introduced in schools during the seven days of testing?
- Did you/your household do anything differently at home during the seven days of testing? Why/why not?
- How did you/your child feel about these changes?

Understanding and impact of test results

- How are test results communicated to you and your child?
- What did you think about the information you received with the test result? Was anything unclear / missing?
- What is your understanding of what the test result means?
- How did you feel when your child received their test result?
- How did it affect you? What were you able to do/not do?
- How did it affect your child? What were they able to do /not do?
- Did you have any concerns about what you/ your child could / could not do?
- Were there any times that it was difficult to follow these rules?
- How confident were you that the results of the test were accurate?

Improvements

- If necessary, would you be happy for your child to take daily tests instead of self-isolating again?
- What could be done to make it easier for people to take tests instead of isolating?
- What could be done to improve daily testing?

**Daily testing sample (staff/teachers)**

I would like to start by asking you about your experiences of daily testing.

- What was your initial reaction when you heard about daily testing as an alternative to self-isolation?
- What made you decide to carry out daily testing at school instead of self-isolating?
- How do you think daily testing compares with self-isolation? Why?
- Did you have any concerns or worries about daily testing?
- How have parents/colleagues/students responded? Are you aware of any concerns others may have?

Experiences of testing

- Can you tell me about the process of daily testing? What happens when pupils take the seven days testing?
- What happens when there is a positive DCT result?
- What is the most difficult /challenging part of daily testing?
- Any groups struggling? ECV?

Behaviour during testing

- What (if any) changes been introduced in the school/classroom during the seven days testing period?
- What (if any) impact has this had on staff? On pupils? On parents?

Communicating test results

- What is your understanding of what the test results mean?
- How are test results communicated to parents and pupils?
- Do you have any concerns about these communications?
- Is there anything missing from the communications?
- What changes (if any) need to be made?

How confident are you that the test results are accurate?

Improvements

- If necessary, would you be happy to take daily tests instead of self-isolating again?
- What could be done to make it easier for people to take tests instead of isolating?
- What could be done to improve daily testing?

**Self-isolating (pupils)**

I would like to start by asking you about your experiences of being a close contact of someone with the virus.

- What happened when you were told that you had been in contact with someone with the virus?
- What made you decide to self-isolate instead of daily testing?
- How did you feel about other people in your class taking tests instead of self-isolating?
- Did you have any concerns or worries about daily testing?
- Did your family/household have any concerns about daily testing?
- Did you have any concerns or worries about self-isolating?
- Did your family/household have any concerns about self-isolation?

Experiences of self-isolation

- Can you tell me about your experiences of having to self-isolate?
- What was the most difficult part of having to self-isolate for you/your family?
- What did you do to overcome any problems you had?
- What would have helped you overcome any problems that you had?

Adherence

There’s loads of guidance to do with self-isolation, and we know people can find some of it tricky

- Can you tell us about any times that you had to leave the house
- Can you tell us about receiving visitors
- What, if anything, did you do differently in the home while you were self-isolating?
- Did you take any extra precautions to reduce infection in the home? Why/why not?

Daily testing in the future

- Would you be willing to take daily tests instead of self-isolating in the future? Why/why not?

**Self-isolating (parents)**

- What happened when you were told that your child had been in contact with someone with the virus?
- What made you decide that your child should self-isolate instead of daily testing?
- Did you have any concerns or worries about daily testing?
- Did you have any concerns or worries about self-isolating?

Experiences of self-isolation

- Can you tell me about your experiences of having a child who was self-isolating?
- What was the most difficult part of having a child self-isolate for you/your family/your child?
- What did you do to overcome any problems you had?
- What would have helped you overcome any problems that you had?

Adherence

There’s loads of guidance to do with self-isolation, and we know people can find some of it tricky

- Can you tell us about any times that your child had to leave the house?
- Can you tell us about any times that you had to receive visitors?
- What, if anything, did you do differently in the home while your child was self-isolating?
- Did you take any extra precautions to reduce infection in the home? Why/why not?

Daily testing in the future

- If necessary, would you be happy for your child to take daily tests instead of self-isolating in the future? Why/why not?
